# Transfer learning with CNNs for efficient prostate cancer and BPH detection in transrectal ultrasound images

**DOI:** 10.1038/s41598-023-49159-1

**Published:** 2023-12-09

**Authors:** Te-Li Huang, Nan-Han Lu, Yung-Hui Huang, Wen-Hung Twan, Li-Ren Yeh, Kuo-Ying Liu, Tai-Been Chen

**Affiliations:** 1 Department of Radiology, Kaohsiung Veterans General Hospital, No. 386, Dazhong 1st Rd., Zuoying Dist., Kaohsiung, 81362 Taiwan; 2 Department of Medical Imaging and Radiological Science, I-Shou University, No. 8, Yida Rd., Jiaosu Village, Yanchao District, Kaohsiung, 82445 Taiwan; 3 Department of Pharmacy, Tajen University, No.20, Weixin Rd., Yanpu Township, Pingtung, 90741 Taiwan; 4 Department of Radiology, E-DA Hospital, I-Shou University, No.1, Yida Rd., Jiao-Su Village, Yan-Chao District, Kaohsiung, 82445 Taiwan; 5 Department of Life Sciences, National Taitung University, No.369, Sec. 2, University Rd., Taitung, 95092 Taiwan; 6 Department of Anesthesiology, E-DA Cancer Hospital, I-Shou University, No.1, Yida Rd., Jiaosu Village, Yanchao District, Kaohsiung, 82445 Taiwan; 7 Institute of Statistics, National Yang Ming Chiao Tung University, No. 1001, University Road, Hsinchu, 30010 Taiwan

**Keywords:** Prostatic diseases, Benign prostatic hyperplasia, Prostate cancer, Machine learning

## Abstract

Early detection of prostate cancer (PCa) and benign prostatic hyperplasia (BPH) is crucial for maintaining the health and well-being of aging male populations. This study aims to evaluate the performance of transfer learning with convolutional neural networks (CNNs) for efficient classification of PCa and BPH in transrectal ultrasound (TRUS) images. A retrospective experimental design was employed in this study, with 1380 TRUS images for PCa and 1530 for BPH. Seven state-of-the-art deep learning (DL) methods were employed as classifiers with transfer learning applied to popular CNN architectures. Performance indices, including sensitivity, specificity, accuracy, positive predictive value (PPV), negative predictive value (NPV), Kappa value, and Hindex (Youden’s index), were used to assess the feasibility and efficacy of the CNN methods. The CNN methods with transfer learning demonstrated a high classification performance for TRUS images, with all accuracy, specificity, sensitivity, PPV, NPV, Kappa, and Hindex values surpassing 0.9400. The optimal accuracy, sensitivity, and specificity reached 0.9987, 0.9980, and 0.9980, respectively, as evaluated using twofold cross-validation. The investigated CNN methods with transfer learning showcased their efficiency and ability for the classification of PCa and BPH in TRUS images. Notably, the EfficientNetV2 with transfer learning displayed a high degree of effectiveness in distinguishing between PCa and BPH, making it a promising tool for future diagnostic applications.

## Introduction

Deep learning methods have gained significant traction in various fields, including medicine, natural sciences, computer sciences, technical sciences, and life sciences^1–4^. Over the past decade, deep learning approaches have been successfully applied in a wide array of fields, such as computed tomography (CT)^5–7^, magnetic resonance imaging (MRI)^8,9^, digital radiography (DR)^9–11^, positron emission tomography (PET)^12–16^, and ultrasound tomography^17–19^. Given their widespread success, deep learning methods have been considered for classifying tasks within ultrasound images.

One area where deep learning techniques have demonstrated promise is in the diagnosis and management of conditions that predominantly affect the aging male population. As life expectancy continues to increase globally, there is a growing need for accurate diagnostic tools and effective treatments for age-related health issues. Prostate cancer (PCa) and benign prostatic hyperplasia (BPH) are two such conditions that are highly prevalent among aging males^20,21^. The early detection of PCa or BPH is crucial for maintaining health and ensuring accurate diagnoses, as timely intervention can significantly improve patient outcomes.

MRI, digital rectal examination (DRE), and transrectal ultrasound (TRUS) are common clinical tools for studying PCa and BPH^22–24^. Among these, TRUS offers several advantages, including no radiation exposure, ease of operation, and real-time scanning. However, false-positive and negative rates hinder diagnostic accuracy due to spike noise, depth attenuation effects, and scattering phenomena between media^25–31^. In light of these challenges, there is considerable interest in exploring the potential of deep learning techniques for improving the diagnostic process.

Deep learning approaches have recently been applied to classify BPH and PCa using MRI. While TRUS remains a popular imaging tool for prostate clinical diagnostic studies, the classification of PCa and BPH using deep learning methods warrants further investigation^32,33^. Some studies have applied deep learning methods to diagnose prostate cancer using B-mode ultrasonography and sonoelastography^32,33^. B-mode ultrasound is potentially suitable for PCa imaging due to its real-time capabilities. As a result, artificial intelligence with deep learning schema has been employed to improve diagnostic accuracy in PCa via B-mode ultrasound (TRUS)^32,33^. Furthermore, machine learning approaches with feature-based techniques continue to progress, enhancing classification performance for PCa^32,33^.

The application of deep learning techniques in the medical field is particularly promising due to the large volumes of data generated by modern imaging techniques. By harnessing the power of big data, researchers can develop more sophisticated algorithms capable of identifying subtle patterns and correlations that might otherwise be missed by conventional diagnostic methods. As such, the use of deep learning methods has the potential to revolutionize the way we approach the diagnosis and management of age-related health issues in the aging male population.

In this context, there is a pressing need for studies that systematically evaluate the performance of deep learning methods in classifying PCa and BPH using ultrasound images. Such investigations can provide valuable insights into the strengths and limitations of various techniques and help guide the development of more effective diagnostic tools. Additionally, by comparing the performance of deep learning methods with that of traditional machine learning techniques, researchers can gain a better understanding of the unique contributions that each approach brings to the table. Artificial Intelligence is a branch of computer science focused on creating systems capable of performing tasks that usually require human intelligence. One powerful approach within AI is deep learning, a subset of machine learning methods based on artificial neural networks with multiple layers. Specifically, we employ CNN models, a type of deep learning model particularly well-suited for analyzing visual data. CNNs use mathematical operations to automatically and adaptively learn spatial hierarchies of features from the input images. In simpler terms, these networks can learn to identify important patterns or features in images—such as textures or shapes—that are indicative of certain conditions like BPH or PCa. The use of transfer learning, another key concept in this study, involves taking a pre-trained CNN model—typically trained on a large general dataset—and fine-tuning it for a specific task, in this case, the diagnosis of BPH and PCa from transrectal ultrasound images. This approach allows us to leverage the power of deep learning without the need for an exceedingly large dataset specific to our medical application.

The primary objective of this study is to investigate and compare the classification performance of PCa and BPH using B-mode ultrasound, based on popular deep learning methods with transferred learning. By exploring the potential of these advanced techniques, we aim to contribute to the ongoing efforts to improve diagnostic accuracy and early detection for both PCa and BPH, ultimately leading to better health outcomes for the aging male population.

## Methods and materials

### Ethics approval

This study was conducted after approval by the Institutional Review Board of Kaohsiung Veterans General Hospital (VGHKS IRB; No. VGHKS13-CT6-04). Due to the retrospective nature of the study, informed consent was waived by the VGHKS IRB. This study was conducted with the methods in accordance with relevant guidelines and regulations.

### The enrolled samples and research flowchart

The cases in PCa and BPH were 1380 and 1530. The age (years), prostate-specific antigen (PSA (ng/mL)), and TRUS images were collected in this work. The TRUS procedures were conducted by a team of three experienced radiologists. As for the prostate biopsy, it involved a combination of a general 12-cores systematic biopsy and targeted biopsies for suspicious lesions. Importantly, the prostate biopsy was also image-guided using sonography, ensuring precise targeting and enhanced diagnostic accuracy. The criteria for selecting TRUS images included focusing on clinically relevant areas, particularly those where biopsies were performed. This selection was guided by the radiologists’ expertise to ensure the relevance and quality of the images used.

The descriptive statistics were shown in Table 1. The mean ± SD (Standard Deviation, SD) of age between BPH and PCa groups were 64.3 ± 9.4 (years) and 68.1 ± 9.8 (years). The mean ± SD of PSA between BPH and PCa were 1.7 ± 0.5 (ng/mL) and 28.7 ± 45.7 (ng/mL). All of the collected TRUS images were initially evaluated by experienced radiologists in an outpatient setting before being subjected to prostate biopsy. These evaluations were conducted prior to the biopsy procedure to identify potential areas of interest. Subsequently, the diagnosis for each image was confirmed via pathological biopsy to classify as either Prostate Cancer (PCa) or Benign Prostatic Hyperplasia (BPH).

**Table 1:** The age and PSA between groups.

Group	Age (years)		PSA (ng/mL)	
	Mean	STD	Mean	STD
BPH(n = 1530)	64.3	9.4	1.7	0.5
PCa (n = 1380)	68.1	9.8	28.7	45.7

Clinically significant prostate cancer is a form of the disease that is likely to grow and spread if left untreated, thereby negatively affecting an individual’s health and life expectancy. This type of prostate cancer is typically characterized by a higher Gleason score, larger tumor volume, extracapsular extension, and the presence of symptoms such as urinary problems and pain, which can impact quality of life. Additionally, Prostate-Specific Antigen (PSA) levels can serve as another indicator. General guidelines categorize PSA levels as follows: low risk (PSA levels below 4.0 ng/mL), moderate risk (PSA levels between 4.0 and 10.0 ng/mL), high risk (PSA levels between 10.0 and 20.0 ng/mL), and very high risk (PSA levels above 20.0 ng/mL). In this study, the optimal Convolutional Neural Network (CNN) method that we presented was used to compare the performance of classifications among these various categorized groups of PSA levels, as reflected in transrectal ultrasound (TRUS) images.

**Figure 1:**
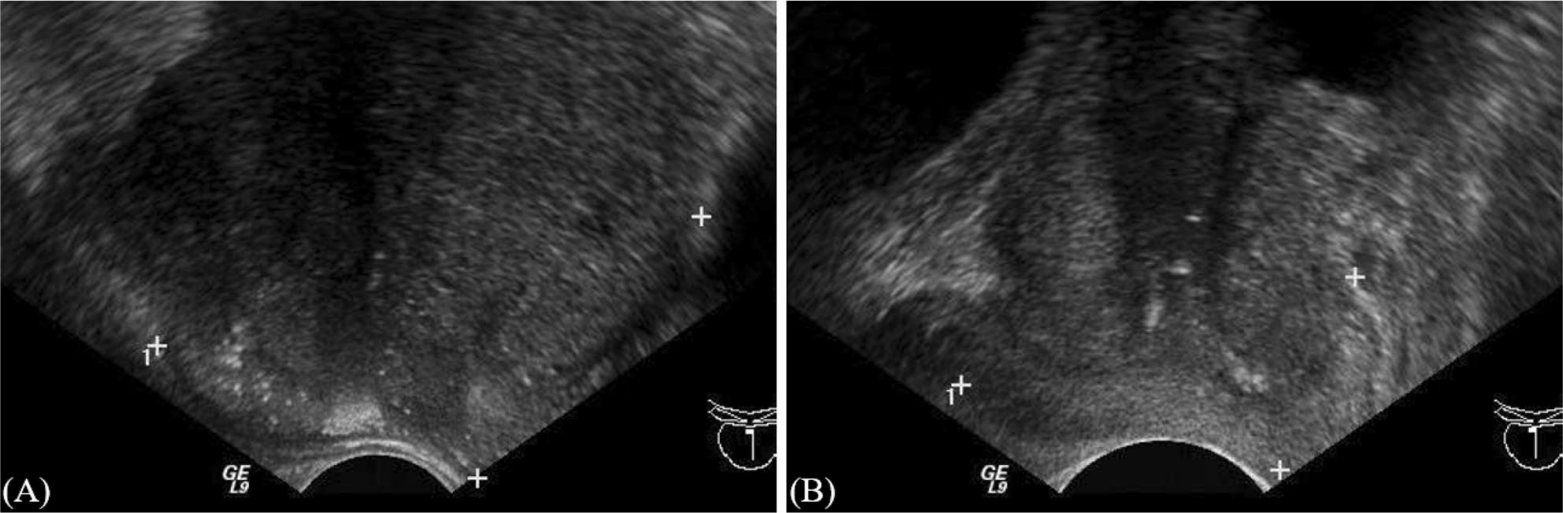
Shows the (**A**) maligned prostate cancer and (**B**) BPH images.

The preprocessing of the TRUS images was necessary before training classification models. In order to remove any identifying information (such as patient name, patient ID, hospital name, and other information) from the input images, the modified images were created by setting intensity zeros outside the prostate area (Fig. 1). The TRUS images were in the size of 640 × 480 with gray-level PNG format. The size and format of the modified images remained the same as the input images. Meanwhile, the extra information might interfere with the classification accuracy using the presented methods. The modified images were created according to the boundary of the dashed line in the input image. The main purpose of creating modified images was to avoid the influences on classification due to the header information around the image (Fig. 1 right). The header information included patient ID, study date, and scanning parameters (i.e., depth ruler, imaging settings, gray level bar, and measurement information).

**Figure 2:**
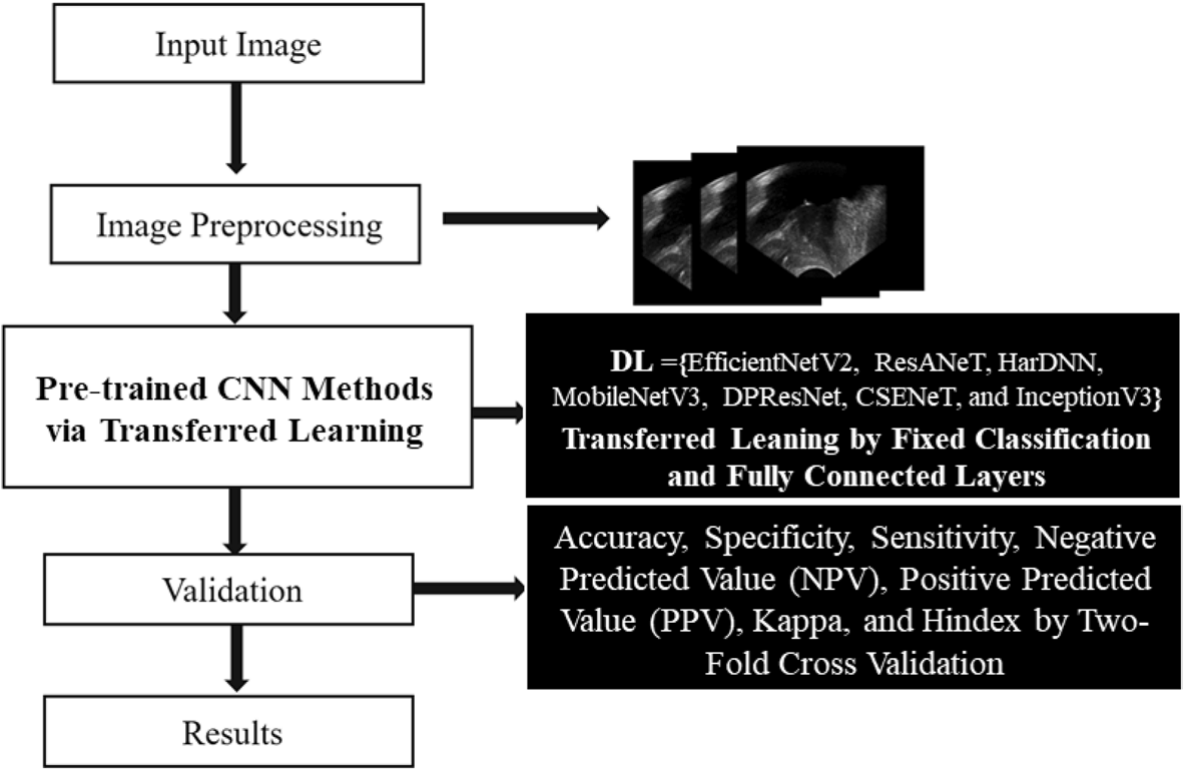
Show the flowchart in this study.

The flowchart in this study included input images, preprocessing, pre-training deep learning methods (DL) via transfer learning, validation, and results (Fig. 2). In the image processing step, the input images were modified by excluding header information and saved as new images. The investigated popular and latest deep learning approaches included seven deep learning methods, as described in “The enrolled samples and research flowchart” section. Next, the investigated models with different parameter settings were validated based on accuracy, specificity, sensitivity, negative predictive value (NPV), positive predictive value (PPV), Kappa, and Hindex values.

### The image processing

The input images were performed the following processing steps before loading into CNN training model. There are three steps were performed for the input image. (1) Resized the images to a uniform dimension of [insert dimensions here], to ensure consistency and reduce computational complexity. (2) Normalized the pixel values of the images to fall within the range [0, 1], improving the numerical stability and convergence speed during model training. (3) Converted grayscale images to RGB by duplicating the grayscale channel to all three RGB channels, as the pre-trained models we employed were originally trained on RGB images.

### The deep learning via transferred learning

The DL methods employed the popular convolutional neural networks (CNN), including feature-map based convolutional neural network (HarDNN)^34^, InceptionV3^35^, MobileNetV3^36^, competitive squeeze and excitation neural network (CSENeT)^37^, residual attention neural network (ResANeT)^38^, deep pyramidal residual neural network (DPResNet)^39^, and EfficientNetV2^40^ via transfer learning (as shown in Supplementary Materials). The investigated CNN methods were regarded as the best deep CNN architectures for classification tasks. Meanwhile, the classification layer and fully connected layer were modified and fixed for two classes (i.e., the original designed number of classes was 1000). Additionally, a batch size of 5 was investigated for the optimal accuracy of the presented methods. The two-fold cross-validation was designed to evaluate the classification performance for the presented methods. In this study, the balance between PCa and BPH cases is close to a 50/50 split. Under these conditions, two-fold stratified cross-validation was chosen as an appropriate method to maintain this balance in each fold of the data.

### Evaluated perofrmance of presented methods

The performance of the presented models was evaluated using the testing set. The testing set consisted of 50% randomly sampled data from each group, with twofold cross-validation. The testing performance of the presented methods is typically assessed using popular indices. A confusion matrix is often employed in the literature to evaluate the suitability of different models, including their sensitivity, specificity, accuracy, positive predictive value (PPV), negative predictive value (NPV), Kappa value, and Hindex index (or Youden’s index).

## Results

The testing results were evaluated based on confused matrix via twofold cross validation. The Table 2 was shown the confused matrix provided by seven transferred learning models. All of the accuracy is over 0.95. Especially, the accuracy provided by HarDNN, MobileNetV3, ResANeT, DPResNet, and EfficientNetV2 are beyond 0.99. These results demonstrate the effectiveness of the CNN models in classifying BPH and PCa using transrectal ultrasound images. Among the seven models, EfficientNetV2 showed the highest accuracy and balanced true positive rates for both BPH and PCa.

**Table 2:** Twofold cross-validation accuracy for prostate cancer classification using seven CNN models.

CNN	TRUE	Predicted as BPH	Predicted as PCa	Accuracy
HarDNN	BPH	459	0	0.9954
	PCa	4	410	
InceptionV3	BPH	436	23	0.9702
	PCa	3	411	
MobileNetV3	BPH	455	4	0.9931
	PCa	2	412	
CSENeT	BPH	449	10	0.9771
	PCa	10	404	
ResANeT	BPH	459	0	0.9954
	PCa	4	410	
DPResNet	BPH	459	0	0.992
	PCa	7	407	
EfficientNetV2	BPH	458	1	0.9977
	PCa	1	413	

Table 3 presents the performance index of the seven CNN models, including accuracy, sensitivity, specificity, PPV (positive predicted value), NPV (negative predicted value), Kappa, and Hindex, sorted in descending order by Hindex. The top-performing CNN based on the maximum Hindex value was EfficientNetV2, which provided an accuracy of 0.9977, sensitivity of 0.9980, specificity of 0.9980, PPV of 0.9976, NPV of 0.9978, and Kappa of 0.9954. The investigated CNN models demonstrated their usefulness and feasibility in performing classification between PCa and BPH sonography. Furthermore, all the investigated CNNs with transfer learning generated outstanding performance, with Hindex and Kappa values of no less than 0.940 in this study. EfficientNetV2 combined neural architecture search and scaling with added optimization. Additionally, the EfficientNetV2 method could provide adaptive regularization and dynamically adjust regularized parameters of training models according to the image size of TRUS B-mode tomography. As a result, the classification performance of EfficientNetV2 was superior to the other investigated methods in this study.

**Table 3:** The performance index provided by seven CNN models were shown the accuracy, sensitivity, specificity, PPV (positive predicted value), NPV (negative predicted value), Kappa, and Hindex with sorting descending by Hindex.

CNN	Accuracy	Sensitivity	Specificity	PPV	NPV	Kappa	Hindex
EfficientNetV2	0.9977	0.9980	0.9980	0.9976	0.9978	0.9954	0.9960
ResANeT	0.9954	0.9903	1.0000	1.0000	0.9914	0.9908	0.9903
HarDNN	0.9954	0.9900	1.0000	1.0000	0.9914	0.9908	0.9900
MobileNetV3	0.9931	0.9950	0.9920	0.9904	0.9956	0.9862	0.9870
DPResNet	0.9920	0.9831	1.0000	1.0000	0.9850	0.9839	0.9831
CSENeT	0.9771	0.9758	0.9782	0.9758	0.9782	0.9541	0.9545
InceptionV3	0.9702	0.9928	0.9499	0.9470	0.9932	0.9404	0.9431

Table 3 displays the performance index of the investigated CNNs under twofold cross-validation with Hindex values greater than 0.940. The maximum accuracy, sensitivity, specificity, PPV, NPV, Kappa, and Hindex were generated by EfficientNetV2 with transfer learning. In contrast, the investigated CNN with the lowest Hindex was InceptionV3, which generated accuracy, sensitivity, specificity, PPV, NPV, and Kappa values of 0.9702, 0.9928, 0.9499, 0.9470, 0.9932, and 0.9404, respectively. Therefore, feasible models should consider both low false positive and negative rates. It is challenging to minimize both false negative and positive rates simultaneously. Thus, the maximized Hindex was considered a feasible index to choose an optimal model in this study. These state-of-the-art and popular CNNs with transfer learning were regarded as suitable for the classification task between PCa and BPH in this study. The optimal investigated CNN was EfficientNetV2 in this study.

Table 4 summarizes the performance of the EfficientNetV2 model in classifying cases of PCa and BPH using TRUS images across different PSA levels. The classifications are further stratified based on PSA levels, broken down into four categories: ≤ 4 ng/mL, 4–10 ng/mL, 10–20 ng/mL, and > 20 ng/mL. For the PCa group with PSA ≤ 4 ng/mL, 1 case was incorrectly classified as BPH, while 119 cases were correctly identified as PCa. In the range of 4–10 ng/mL, 3 cases of PCa were misclassified as BPH, and 437 were correctly classified as PCa. For the PCa group with PSA between 10 and 20 ng/mL, 6 cases were wrongly identified as BPH, and 469 were accurately classified as PCa. In the PCa group with PSA > 20 ng/mL, 2 cases were misclassified as BPH, whereas 343 cases were correctly identified as PCa. For the BPH group with PSA ≤ 4 ng/mL, 1525 cases were correctly classified, and 5 were wrongly identified as PCa. This table elucidates the EfficientNetV2 model capability in classifying PCa and BPH conditions from TRUS images, providing valuable insights into the model's performance at different PSA level strata. The table reveals that EfficientNetV2 performs remarkably well, especially at lower PSA levels for BPH and across all PSA categories for PCa. However, there were minor instances of misclassification. Hence the model shows promise as a diagnostic tool when used in conjunction with PSA levels.

**Table 4:** Classification performance of EfficientNetV2 in identifying prostate cancer (PCa) and benign prostatic hyperplasia (BPH) across different PSA levels.

TRUE	Predicted	PSA ≦ 4	4 < PSA ≦ 10	10 < PSA ≦ 20	PSA > 20	Total
PCa	BPH	1	3	6	2	12
	PCa	119	437	469	343	1368
BPH	BPH	1525	NA	NA	NA	1525
	PCa	5	NA	NA	NA	5

NA indicates that all BPH cases in this study had PSA levels less than 4 ng/mL. Therefore, no BPH cases were present in the other PSA level categories.

## Discussion

### Comparisions between presented results and published articles

Deep learning techniques have played an essential role in diagnosing PCa over the past decade^27^. In recent articles^27–29^, magnetic resonance imaging (MRI) has been commonly used and studied for the segmentation, classification, and detection of PCa in computer-aided diagnosis with artificial intelligence methods. However, only a few deep learning tools have been utilized for classifying PCa with TRUS images. Therefore, the primary objective of this study was to classify PCa with TRUS images and investigate the classification performance of PCa using popular CNNs with transfer learning. The analytical results showed feasible and reasonable classification between PCa and BPH. The EfficientNetV2, ResANeT, HarDNN, MobileNetV3, DPResNet, CSENeT, and InceptionV3 with transfer learning are useful deep CNN methods for classifying TRUS images. Furthermore, all the accuracy, specificity, sensitivity, PPV (also called Precision), and Hindex values were higher than 0.940, as demonstrated by the investigated CNN methods in this study. The presented methods showcased their ability and efficiency.

Published results using CNN or SVM are listed in Table 5^17,30,41–44^. Feature-based classification between PCa and BPH with ultrasound images has commonly employed SVM classifiers^17,30,42^. However, generating useful features from images remains a challenge. In contrast, CNNs have been applied to B-mode ultrasound to create an efficient end-to-end approach^27^.

**Table 5:** The presented method was compared with the public methods.

Authors	Year	Classification^†^	Method	Modality	Performance (%)*
Zhang et al.^30^	2020	47 vs 56	SVM	B-Mode US	87.9/87.0/88.8
Feng et al.^41^	2018	7511 vs 25,738	cnn	CEUS	90.2/82.8/91.5
Zhiyong et al.^42^	2021	66 vs 103	r-cnn + xception	TRUS	80.4/92.4/72.8
Huang et al.^17^	2020	130 vs 126	SVM	B-Mode US	70.9/70.0/71.7
Imani et al.^43^	2015	625 vs 576	SVM	MRI + RF-ultrasound	80.0/88.0/80.0
Li et al.^44^	2023	100 vs 136	Deep learning	MRI	88.9/83.9/92.7
This work	2022	1380 vs 1530	EfficientNetV2	TRUS	99.7/99.8/99.8

^†^Means the sample size between malignant (positive) and benign (negative or BPH).

*Means accuracy/sensitivity/specificity. CEUS means the contrast-enhanced ultrasound. B-Mode US means brighten mode ultrasound. RF-Ultrasound means radio frequency ultrasound.

Feature-based classification of PCa using ultrasound or contrast-enhanced ultrasound has been conducted by Zhang^30^, Huang^17^, Imani^43^, and Li^44^. The highest accuracy, sensitivity, and specificity among these results are 87.9%, 88.0%, and 88.8%. Moreover, deep learning methods have been applied to classify PCa using prostate ultrasound by Feng^41^ and Zhiyong^42^. The highest accuracy, sensitivity, and specificity among these results are 90.2%, 92.4%, and 91.5%. CNN improves the classification performance between PCa and benign prostate ultrasound compared with feature learning methods.

The accuracy, sensitivity, and specificity achieved by EfficientNetV2 in this study are 99.4%, 99.2%, and 99.5%, respectively (Table 5). Transfer learning via CNNs yields satisfactory and acceptable classification results. Indeed, the methods presented in this study utilize state-of-the-art CNN techniques to create a useful model for classifying TRUS images with feasible performance.

### Using the state-of-art pre-trained CNNs

In this study, the performance of seven state-of-the-art pre-trained CNNs was investigated for classifying TRUS images. The developed architectures of these latest CNN models were based on directed acyclic graph (DAG) networks^34–40^. A DAG network has a more complex architecture, designed from multiple layers. DAGs possess a rich assortment of algorithms needed for non-linear steps in the complicated geometry of multiple CNNs.

A DAG network has useful properties, including reachability, transitive closure, and transitive reduction. As a result, any DAG can quickly optimize and handle multiple layers as input, as well as output from multiple layers. Meanwhile, DAG algorithms are merited for searching the shortest path for designing nodes (or architectural layers). This is one of the reasons why the investigated CNNs in this study could provide high performance classification for TRUS images.

However, several parameters used in CNNs can affect the classification performance, including input image size, batch size, number of epochs, learning rates, loss function, and optimizer. Therefore, setting the parameters of a CNN model is a crucial and essential step in building a useful CNN model.

### Efficient detection between BPH and PCa

The success of these CNN models can be attributed to their ability to adapt to the unique characteristics of TRUS images and the utilization of transfer learning. This approach allowed the models to leverage pre-existing knowledge from large-scale datasets, enabling them to perform better in the classification task. The promising results suggest that the investigated CNNs, particularly the EfficientNetV2, hold great potential as reliable tools for accurately classifying TRUS images and distinguishing between BPH and PCa cases. Furthermore, these findings contribute to the growing body of research on the application of deep learning techniques in medical image analysis, particularly for prostate cancer diagnosis. The use of efficient and accurate deep learning models can significantly improve the clinical decision-making process and ultimately lead to better patient outcomes. By employing state-of-the-art CNNs for the classification of TRUS images, this study has demonstrated the potential of these methods in enhancing the detection of BPH and PCa, which is essential for effective treatment planning and management. Therefore, the added values of incorporating Convolutional Neural Network (CNN) architectures into our study are summarized as below.

1. *Improved accuracy* Although PSA levels are valuable, they are not infallible indicators. Elevated PSA levels can also result from other conditions like prostatitis or even urinary tract infections. Our CNN model aims to improve diagnostic accuracy when used in conjunction with PSA levels.
2. *Clinically significant PCa* PSA levels can indeed differentiate PCa from BPH to some extent but may not be as effective in identifying clinically significant PCa from clinically insignificant forms. Our CNN model seeks to offer additional parameters for more nuanced differentiation.
3. *Threshold ambiguity* While general guidelines for PSA levels (e.g., low, moderate, high risk) exist, there are often “gray zones” where PSA levels are inconclusive. Our CNN model could provide additional confidence in these borderline cases.
4. *Early detection* Lower PSA levels may not always guarantee the absence of cancer, especially in its early stages. CNN models trained to detect subtle changes in TRUS images might provide an additional layer of security in early detection efforts.
5. *Reducing invasive procedures* If the CNN model can reliably identify BPH or clinically insignificant PCa, it may reduce the need for more invasive diagnostic procedures like biopsies, thus reducing healthcare costs and patient discomfort.

### Summary

This study explored the efficacy of seven cutting-edge CNN models, including EfficientNetV2, for classifying PCa and BPH through TRUS images. Given the crucial importance of early detection of PCa and BPH for the health and well-being of aging men, this paper bridges the gap between medical diagnostics and visual sciences.

We took special care to remove header information from the TRUS images to avoid any interference during the CNN-driven feature extraction process. This step proved to be critical for the successful application of our CNN algorithms. Utilizing a twofold cross-validation approach, we achieved impressive results—specifically, an accuracy of 0.9977, sensitivity of 0.9980, and specificity of 0.9980.

Among the models evaluated, EfficientNetV2 stood out for its effectiveness and efficiency in classifying TRUS images, thus distinguishing between PCa and BPH. This research demonstrates the synergistic potential of combining deep learning and machine learning techniques to achieve high diagnostic accuracy. Such methods can serve as valuable tools for clinicians, aiding in their diagnostic evaluations and ultimately leading to better treatment plans and improved patient outcomes.

In conclusion, this study underscores the promise that state-of-the-art CNN models, particularly EfficientNetV2, hold in enhancing the diagnostic accuracy of TRUS imaging for PCa and BPH. These findings have significant implications for the medical community, especially in ensuring the timely and accurate diagnosis crucial to the health and well-being of an aging male population.

### Limitations and future works

While this study’s results are promising, several limitations warrant discussion, and they naturally suggest avenues for future research.

Firstly, although we utilized transfer learning in the study, alternative pre-training and fine-tuning approaches could be examined to potentially augment the CNN models’ performance. Also, the design of task-specific architectures tailored to TRUS-based classification of PCa and BPH may yield improved results.

Secondly, the issue of interobserver bias cannot be overlooked. Different radiologists might interpret TRUS images in varying ways, introducing a level of variability that challenges the development of a universally effective AI model. While the images used in our study were initially evaluated by seasoned radiologists, a multi-center study involving diverse professionals could help to further validate the robustness of our AI-driven approaches. The image selection might impact the results and to assure that the selected images correlate with the biopsy areas.

Future research could delve into integrating multi-modal data, like combining TRUS images with MRI scans, to improve model performance. Methods to better understand and visualize the CNN models’ learned features could increase trust among medical professionals, aiding in more widespread adoption. The development of real-time classification systems, capable of providing immediate feedback during clinical assessments, is another potential area for future work. Longitudinal studies could also offer valuable insights into the natural history and treatment effectiveness for PCa and BPH.

By addressing these limitations and venturing into these future research areas, we believe the utility of CNN models for classifying PCa and BPH through TRUS images can be significantly enhanced, thus contributing to more precise and timely diagnostic processes, as well as improved patient outcomes.

### Supplementary Information

Supplementary Information.

## Data Availability

The data presented in this study are available upon request from the corresponding author. The data are not publicly available due to restrictions, e.g., privacy and ethical concerns.

## Abbreviations

AI: Artificial intelligent
BPH: Benign prostatic hyperplasia
CNN: Convolutional neuron networks
CSENeT: Competitive squeeze and excitation neural network
CT: Computed tomography
DAG: Directed acyclic graph
DL: Deep learning
DPResNet: Deep pyramidal residual neural network
DR: Digital radiography
HarDNN: Feature-map based convolutional neural network
Hindex: Youden’s index
MRI: Magnetic resonance imaging
NPV: Negative predictive value
patient ID: Patient identification
PCa: Prostate cancer
PET: Positron emission tomography
PPV: Positive predictive value or precision
PSA: Prostate-specific antigen
ResANeT: Residual attention neural network
STD: Standard deviation
TRUS: Transrectal ultrasound
