# Supplementary material for: Transfer learning with CNNs for efficient prostate cancer and BPH detection in transrectal ultrasound images
Source: Sci Rep. 2023 Dec 9;13:21849. doi: 10.1038/s41598-023-49159-1 (PMC10710441; doi:10.1038/s41598-023-49159-1)
Supplement: Supplementary file 1 — Supplementary Information. [file 41598_2023_49159_MOESM1_ESM.docx]

Supplementary Materials

Table S1 provides a comprehensive overview of the merits of various Convolutional Neural Network (CNN) architectures recently explored in the literature. This table enumerates seven distinct CNNs, accompanied by details about their authors, the year they were introduced, and their unique advantages. For example, HarDNN focuses on accurately estimating feature maps and claims to have computational speeds up to ten times faster than other CNNs. On the other hand, EfficientNetV2 is praised for its efficient training capabilities and for outperforming previous models on a variety of datasets. These CNNs offer different design philosophies and strengths, thereby providing a range of options suitable for diverse applications. The table is designed to serve as a quick reference guide to assist researchers in understanding the unique attributes of each CNN, aiding in the selection of an appropriate architecture for specific research needs.

Table S1. The merits of investigated CNNs in recently.

| CNN | Authors | Year | Merits |
| --- | --- | --- | --- |
| HarDNN | Mahmoud et. al. [35] | 2020 | The HarDNN was accurately estimated a feature map in CNN. HarDNN was demonstrated to improve computational times by 10x faster than other CNNs. |
| InceptionV3 | Kin N. [36] | 2019 | The InceptionV3 was proposed to prompt the accuracy of recognition by performing a fine-tuned parameter in the layers of CNN with transferred learning pre-trained model. The InceptionV3 was trained and validated on a cooking dataset. The InceptionV3 was provide a potential solution to the recognition problem. |
| MobileNetV3 | Chu et. al. [37] | 2019 | MobileNet was used the depth-wise separable convolutions to create a small weight CNN. The global hyper-parameters of MobileNet that efficiently balance between latent parameters and classification accuracy by evaluating ImageNet classification. Meanwhile, MobileNet had demonstrated the across a wide range of applications including object detection, fine-grain classification, face attributes and large-scale geo-localization. |
| CSENeT | Hu et. al. [38] | 2018 | The competitive squeeze-excitation residual network (CSENet) was built for to determined and to expand the meaning of channel relationship in residual layers. The CSENet was proven as good as the popular CNN. |
| ResANeT | Wang et. al. [39] | 2017 | The residual attention network (ResANeT) was built according stacking attention modules with generating attention-aware features. The ResANeT not only be used to train deep residual attention network, but could be easily adjusted to hundreds of layers. |
| DPResNet | Han et.al. [40] | 2016 | The deep pyramidal residual neural network (DPResNet) was designed to ensure effective performance by increasing the diversity of high-level features in an image. The DPResNet had been proven to improve generalization ability to prompt the classification accuracy. |
| EfficientNetV2 | Tan et.al. [41] | 2021 | The EfficientNetV2 was a new CNN that trains faster and efficiency parameters than other CNN models. The combination of neural architecture searching and scaling with adding optimization. The EfficientNetV2 significantly outperforms previous models on ImageNet and CIFAR/Cars/Flowers datasets. |
